# YeastFab: the design and construction of standard biological parts for metabolic engineering in *Saccharomyces cerevisiae*

**DOI:** 10.1093/nar/gkv464

**Published:** 2015-05-08

**Authors:** Yakun Guo, Junkai Dong, Tong Zhou, Jamie Auxillos, Tianyi Li, Weimin Zhang, Lihui Wang, Yue Shen, Yisha Luo, Yijing Zheng, Jiwei Lin, Guo-Qiang Chen, Qingyu Wu, Yizhi Cai, Junbiao Dai

**Affiliations:** 1 MOE Key Laboratory of Bioinformatics, School of Life Sciences, Tsinghua University, Beijing 100084, China; 2 School of Biological Sciences, The King's Buildings, University of Edinburgh, Edinburgh EH9 3BF, United Kingdom; 3 Wuxi Qinglan Biotechnology Inc., Yixing, Jiangsu 214200, China

## Abstract

It is a routine task in metabolic engineering to introduce multicomponent pathways into a heterologous host for production of metabolites. However, this process sometimes may take weeks to months due to the lack of standardized genetic tools. Here, we present a method for the design and construction of biological parts based on the native genes and regulatory elements in *Saccharomyces cerevisiae*. We have developed highly efficient protocols (termed YeastFab Assembly) to synthesize these genetic elements as standardized biological parts, which can be used to assemble transcriptional units in a single-tube reaction. In addition, standardized characterization assays are developed using reporter constructs to calibrate the function of promoters. Furthermore, the assembled transcription units can be either assayed individually or applied to construct multi-gene metabolic pathways, which targets a genomic locus or a receiving plasmid effectively, through a simple *in vitro* reaction. Finally, using β-carotene biosynthesis pathway as an example, we demonstrate that our method allows us not only to construct and test a metabolic pathway in several days, but also to optimize the production through combinatorial assembly of a pathway using hundreds of regulatory biological parts.

## INTRODUCTION

Natural products have long been used to develop drugs to combat disease and improve human health. One example is the antimalarial drug artemisinin, which is naturally produced by a plant, *Artemisia annua* and has been used for a long time in traditional Chinese medicine (1,2). However, due to the long production time and usually low titre of these products in the original hosts, the imbalance between supply and demand inevitably drives the price of these drugs to a very high level. Therefore, inexpensive biosynthesis of these high value products through metabolic engineering in a heterologous host becomes economically attractive. In the past 20 years, much of the work has been done using the bacterium *Escherichia coli* for chemical production because it is fast growing, easy to manipulate and has mostly characterized metabolic and regulatory pathways (3–5). Recently, interest has begun to shift from *E. coli* to eukaryotes due to *E. coli’*s lack of post-translation modification (6) and the difficulty of expressing complex enzymes such as cytochromes P450 (7,8) in the bacterial system. Needless to say, many natural products such as terpenes are also likely to be anti-bacterial.

The budding yeast *Saccharomyces cerevisiae* is one of the best-studied eukaryotic model organisms and has a long history in the production of fermented beverages, ethanol and other commodities (4,9,10). It has several advantages as a commercial production host: (i) well-established protocols and methods to modify its genome content; (ii) great tolerance to changes of environmental conditions such as temperature, pH and osmotic stress; (iii) formation of diploid cells for robust growth and increased adaptation; (iv) proper post-translational modifications such as glycosylation and disulfide bond formation. Recently, Keasling *et al*. used synthetic biology and metabolic engineering approaches to allow the industrial production of artemisinic acid using *S. cerevisiae* with a production capacity of 25 g/l, much higher than what could be achieved in *E. coli* (11–13).

The vast majority of metabolic pathways to produce a natural product require multiple genes to function coordinately under the control of a particular regulatory network. For example, the nitrogen fixation pathway in *Klebsiella oxytoca* includes a total of 20 genes in seven operons, with probably more regulatory elements that remain unknown (14,15). Therefore, it is very challenging and time-consuming to reconstitute and optimize the entire metabolic pathway in a heterologous system, even when the pathway is fully defined. On the other hand, despite the fact that we are now able to synthesize the entire chromosome and even genome (16,17), it remains beyond the affordability of many research groups to test multiple designs simultaneously. Various technologies have been developed to assemble small DNA fragments into large modules, including restriction enzyme-dependent methods such as Golden Gate assembly (18,19) and restriction enzyme-independent methods such as Gibson assembly (20), CPEC (21), MODAL (22) and DNA assembler (23). These methods have provided the synthetic biology community with great tool kits to quickly generate large DNA constructs. However, all these methods require module-specific custom design, in which parts are not readily to be reused and a lot of re-factoring is inevitable. Furthermore, the development of standardized and well-characterized biological parts greatly facilitates the modular design of biological systems, as exemplified by the MIT Registry of Standard Biological Parts (24–29). However, the MIT Registry largely focuses on prokaryotic systems.

To address these issues, we started with the modular design of standard biological parts in *S. cerevisiae* by defining three classes of parts: the promoters (PRO), open reading frames (ORF) and terminators (TER). The Golden-Gate assembly method was adopted since it is not only relatively inexpensive and highly efficient, but also allows ordered assembly of biological parts according to predefined genetic rules (18,19). Three vectors were designed to host the three types of parts, designed in such a way that a part can be cloned and released using two different type IIs enzymes, namely BsaI and BsmBI, but with the same sticky ends. We demonstrated that the cloning of these parts could be performed with high efficiency and in a high-throughput manner. Next, we used a reporter construct to characterize a subset of these promoters under various growth and stress conditions. In addition, we optimized the assembly of the transcription units (TUs) to combine the PRO, ORF and TER together, and demonstrated that nearly 100% efficiency could be achieved. The expression of each TU could be easily tested by transforming the assembled plasmid into yeast. Furthermore, our hierarchal design allows multiple TUs to be assembled and integrated into a target locus in yeast directly through a simple *in vitro* reaction. Using the β-carotene biosynthesis pathway, we demonstrated that the complete assembly process starting from the cloned parts could be finished within a week with good efficiency. Finally, we performed an unbiased test by generating all permutation with three different promoters to drive the three genes (a total of 27 combinations). We showed that several optimal combinations could be identified. Furthermore, using ∼150 randomly chosen promoters with different strength, we developed a combinatorial assembly protocol to generate a complex yeast library for β-carotene production. Together, we demonstrated the YeastFab standard biological parts and assembly method presented in this study could be widely applied in synthetic biology applications in this model eukaryotic system.

## MATERIALS AND METHODS

### Strains, plasmids and growth conditions

The yeast strain JDY52 (*MATa his3Δ200 leu2Δ0 lys2Δ0 trp1Δ63 ura3Δ0 met15Δ0*), which is derived from S288C, was used as the host for exogenous pathways. The promoter activity was tested in BY4741 (*MATa his3Δ1 leu2Δ0 ura3Δ0 met15Δ0*) (30). The part-accepting vectors (hereafter referred as part vectors) were derived from pSMART HCKan (Lucigen Corporation, Middleton) and the POT vectors were constructed by modifying the pRS plasmids (31). The reporter plasmid to measure promoter activity was based on pPAL1-His3core (32). Standard methods were used to culture and manipulate the yeast strains unless otherwise mentioned. The primers and plasmids used in this study were listed in Supplementary Table S1.

### Unique recombination regions

The URRs were generated using Random DNA Sequence Generator (http://www.faculty.ucr.edu/∼mmaduro/random.htm) with 50% GC content followed by removal of unwanted restriction enzyme sites using GeneDesign 3.0 (33). The sequences were analysed using nucleotide blast (http://blast.ncbi.nlm.nih.gov/Blast.cgi), which indicated there are no sequence homologs from any known sequence databases. The DNA sequences for both URRs are listed in Supplementary Table S1.

### High throughput assembly of parts

Primers used to amplify the parts were synthesized and distributed in 96-well plates. PCR was performed with the standard reaction conditions and the following program: 94°C for 30 s; 5 cycles of 94°C for 30 s, 45°C for 30 s, 68°C for 2 min; 5 cycles of 94°C for 30 s, 50°C for 30 s, 68°C for 2 min; 20 cycles of 94°C for 30 s, 55°C for 30 s, 68°C for 2 min; followed by 68°C for 7 min. The high-fidelity polymerase, KOD (Takara Inc.) was used to diminish possible PCR errors. The ‘one-pot’ reaction was carried out using purified PCR products and part accepting vectors with the following composition (for 10 μl mixture): 1 μl 10× T4 ligase buffer, 0.1 μl 100× bovine serum albumin (BSA), 10 ng acceptor vector, 2 μl PCR product, 4 U of BsaI (NEB), 0.5 U T4 DNA ligase (Thermo Scientific) and appropriate ddH_2_O. The reaction mixture was put into a thermocycler to run the following program: 37°C for 1h, 50°C for 15 min, 80°C for 15 min and finally keep at 10°C. After the reaction, the 5 μl of mixtures were transformed into competent *E. coli* DH5α and selected on LB medium containing 30 μg/ml kanamycin. Two white clones were randomly isolated and subjected to PCR to confirm the correct insertion. The PCR-confirmed clones were sequenced to ensure 100% accuracy of inserted parts.

For those parts with internal restriction sites, the following alternative program was used: 37°C 5 min, 37°C 5 min followed by 25°C 5 min for 3 cycles, 50°C 5 min, 80°C 5 min, then addition of 0.5 U T4 DNA ligase and incubation at 25°C for another 40 min before transforming bacteria.

### Assemble transcription units

In a 10 μl reaction mixture, three types of genetic parts in plasmids (PRO, ORF and TER) and one POT receiving vector were mixed together with 5 U of BsmBI (NEB) in 1× T4 ligase Buffer (Thermo Scientific) and incubated at 55°C for 1 h. Then 0.5 U of T4 DNA ligase (Thermo Scientific) was added into the mixture. A subsequent ligation-heat inactivation program was performed as 25°C 1 h, 50°C 5 min, 80°C 10 min. After reaction, 5 μl mixture was directly transformed into DH5α competent cells and selected on LB containing 50 μg/ml carbenicillin. Generally, at least four white colonies were randomly picked for verification by colony PCR. The PCR-verified clones were further analysed by restriction enzyme digestion.

### Reporter plasmid construction

Similar to the part accepting vectors, the reporter plasmid was re-engineered to contain a bacterial expressed RFP gene for colony selection just upstream of the YFP ORF, which could be released with BsaI, leaving overhangs compatible with promoter parts. The method used was similar to that for part assembly. 1.5 μl PCR fragments and 15 ng reporter plasmids were mixed together with 3 U of BsaI-HF (NEB), 0.5 U of T4 DNA ligase (Thermo Scientific) in 1× T4 ligase Buffer (Thermo Scientific). The same program: 37°C 5 min, 50°C 15 min, 80°C 15 min was used. 1.5 μl of reaction mixture was transformed into bacteria. The oligos YGO199 (GCGTATATATACCAATCTAAGTCT) and YGO200 (GTCAATTTACCGTAAGTAGCATC) were used as primers to set up colony PCR after transformation. The plasmid DNA from positive colony PCR candidates was isolated and sequenced. The correct reporter constructs were transformed into the yeast strain following the standard protocol and selected on synthetic complete medium lacking leucine (SC-Leu). Two independent clones were tested.

### Flow cytometry analysis of promoter activity

The promoter activity was measured by quantifying the fluorescent intensity of yellow fluorescent protein (YFP) and mCherry using an LSRFortessa cell analyser (BD Biosciences) with a HTS automatic sampler. Cell were inoculated into synthetic complete medium lacking Leucine (SC-Leu) in 96-deep-well plates (containing 1 ml of liquid medium) and grown overnight in a 30°C plate shaker. Cells were then diluted in fresh SC-Leu medium (OD_600_ at ∼0.1) and cultured for another 8 h (OD_600_ at ∼1, log phase) before measurement. The 488 nm laser and 530/30 nm filter were used for detection of YFP fluorescent intensity while 561 nm laser and 610/20 nm filter for mCherry. At least 10 000 cells were recorded from each well, among which mCherry positive cells with appropriate size were gated for calculation using FlowJo (version 7.6.1, TreeStar, Supplementary Figure S2). The ratio of YFP fluorescence intensity/mCherry fluorescence intensity was calculated for each cell and the average value of the whole cell population was obtained. For each promoter, two independent clones were measured separately and the mean value was generated, which represents the absolute activity of a given promoter. Furthermore, to compare the measured values among different plates and batches, a strain containing the constitutive pCYC1 promoter was included in every 96-well plate. The abovementioned mean value of every promoter was normalized to that of pCYC1 and used to represent the relative activity of a promoter.

To test promoter activity under stress conditions, cells at log phase were washed twice with sterile water and resuspended in a different medium (ddH2O, SC-Leu with 0.015% H_2_O_2_, SC-Leu lacking glucose, SC-Leu lacking nitrogen (YNB-AA-AS, 2% glucose, 0.025% ammonium sulfate)) (34), or incubated at 37°C for heat treatment. The fluorescence value was measured at 3 or 6 h post-treatment. The activities of every promoter measured in this study were included in Supplementary Table S2.

### Western blotting

The yeast cells containing either an individual gene expressed in a POT vector or multiple genes integrated into the genome were used to detect protein expression. Five minutes alkali treatment and subsequent 10 min boiling in the sodium dodecyl sulfate-polyacrylamide gel electrophoresis (SDS-PAGE) sample buffer (35) were used for protein extraction from yeast. Proteins were then separated on a 12.5% SDS-PAGE gel and transferred to a nitrocellulose membrane. 1:2000 dilution of mouse monoclonal anti-HA (Sigma H3663) and rabbit anti-FLAG (Sigma F7425) antibody were used to detect expression of the relevant tagged components, respectively.

### Assembly of metabolic pathways

The POT vectors containing the assembled TUs, the plasmids containing URRs and a yeast selective marker (LEU2) were digested with restriction enzymes, assembled and transformed into JDY52. The cells were selected on SC-LEU and incubated at 30°C for 2 days before replicating. The candidate clones which could grow on SC-LEU but not SC-HIS were isolated and patched onto a fresh SC-LEU plate. The genomic DNA from these clones was isolated and subjected to PCR analysis to confirm the correct integration of all three TUs at the target locus. To determine the promoter upstream of each gene, primers were designed to amplify the promoter regions and the promoters were amplified and sequenced. The identity of the promoter was determined using the Basic Local Alignment Search Tool (BLAST) tool at Saccharomyces Genome Database (SGD) (http://www.yeastgenome.org/cgi-bin/blast-sgd.pl). The identity and activity of the promoters in each construct was listed in Supplementary Table S3.

### Carotenoid assay

The yeast strains were grown in 5 ml of YPD (220 rpm) at 30°C for 72 h. 4 x 10^8^ cells were collected by centrifugation at 12 000 rpm for 1 min and washed twice with deionized water. The cell pellet was lyophilized in a freeze drier (CHRIST; Alpha 1–2 LD). Dried cells, 0.5 ml 0.5-mm glass beads and 1 ml of 90% acetone were combined in a 2-ml round bottomed plastic tube, which was shaken vigorously in a Mini-Beadbeater-1 (Biospec) operated over 10 cycles of running for 40 s and pausing for 20 s. The cell suspension was transferred to another tube to remove the glass beads, shaken vigorously at 4°C for 10 min, and centrifuged at 14 680 rpm for 10 min, after which the supernatant was collected as the extract. The extracts were dried under reduced pressure in a centrifugal vaporizer (Eppendorf AG 22331; Hamburg) and dissolved in 20 μl hexane. Ten microliters of the 80 μl extracts, which were diluted with 20 μl Hexane/isopropanol (7:3 by volume) and 40 μl Methanol/isopropanol (7:3 by volume; solvent A) were run on a ZORBAX Extend-C18 column (2.1 × 100 mm, 3.5 μm particle size, Agilent Technologies) on an Agilent 1260 HPLC system with the following method (solvent B is ddH_2_O): start at 85% A; hold at 85% A for 1 min; 0.125%/s to 100% A; hold at 100% A for 17 min; 2.5%/s to 85% A; hold at 85% A for 10 min. The absorbance was measured with a diode-array (DAD) UV-Vis detector at 450 nm. Peaks were identified by comparison with the authentic β-carotene (Sigma–Aldrich, C4582) and estimated by integrating peak areas.

## RESULTS

### Overall design for the assembly of a given metabolic pathway

**Figure 1.**
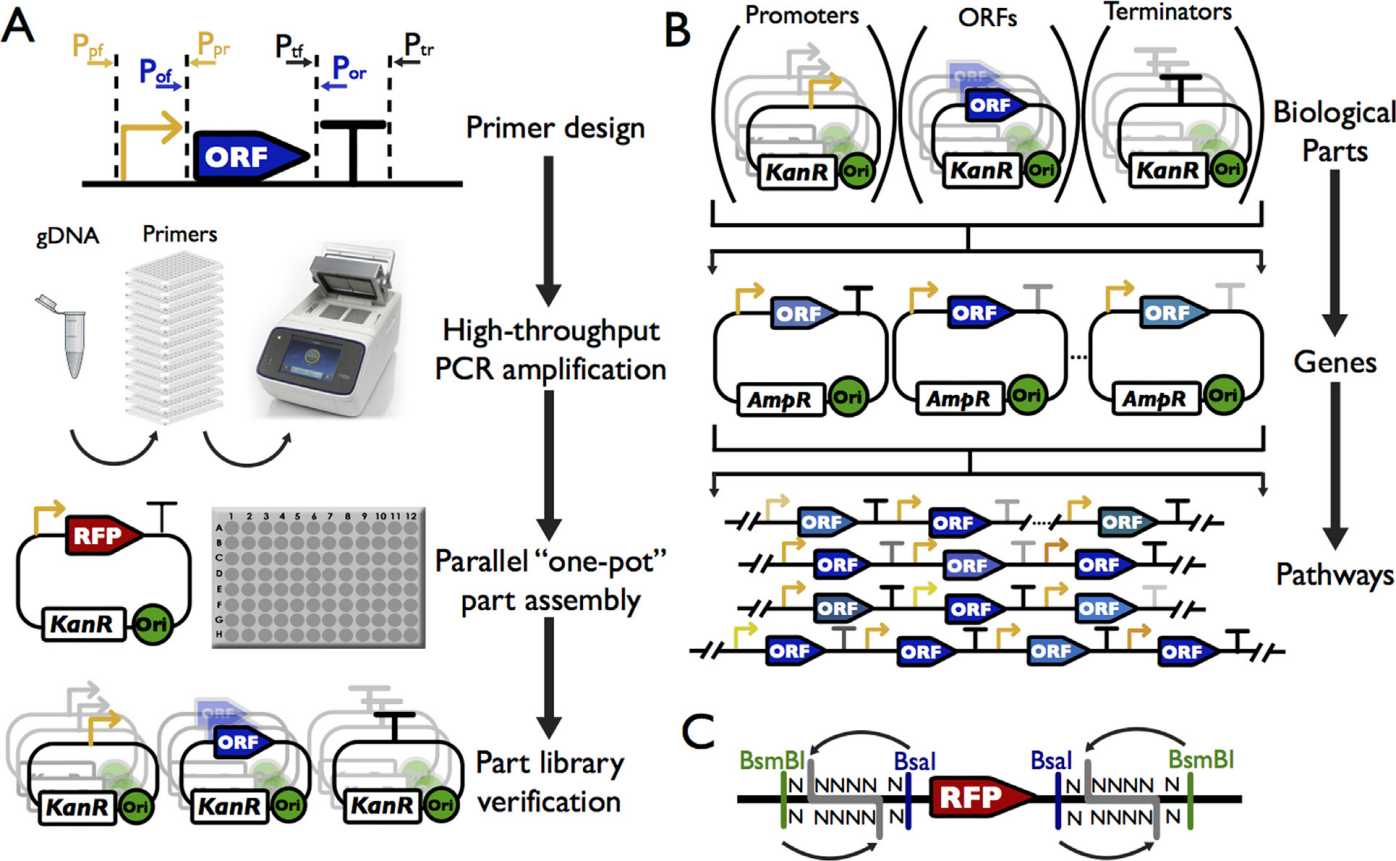
Overall scheme to construct standard biological parts, transcription units and pathways. (**A**) Overall strategy to construct the standard biological parts and to profile their functions. All parts generated in this study are derived from native sequences, amplified from *S. cerevisiae* genome by PCR. Each part is verified by sequencing. (**B**) Use of the part libraries to assemble transcription units (TUs) and pathways. Each part within a library is compatible with the parts from other libraries, allowing compositional assemblies. The TUs can be used for a second round of assembly, leading to the construction of multiple-gene pathways. The assembled pathways can be integrated into either a designated genomic locus or a plasmid. (**C**) Schematic representation of the acceptor vectors for parts. Each vector contains two different type IIs restriction enzyme recognition sites. BsaI was used to release the RFP marker, allowing quick identification of the correctly assembled parts. BsmBI was used to put different parts together to construct the transcription units.

Figure 1 shows the overall workflow to generate, characterize and utilize the standardized biological parts for the assembly of a given metabolic pathway. First, three categories of biological parts will be generated, covering the whole yeast genome. Each part will be amplified from the yeast genome using a pair of specific primers carrying standardized overhangs. Second, the biological function and the regulation under different conditions of these parts will be profiled and used as reference for metabolic engineering. Third, every part from the three libraries can be cherry-picked to assemble a transcription unit (TU), for which the expression level can be predicted and controlled. Fourth, each assembled TU can be used in a second round of assembly, leading to the construction of multiple-gene pathways *in vitro*. Finally, the assembled multiple-gene pathways can be targeted either into a plasmid or integrated into a yeast chromosome for functional testing.

### Design and construction of the standard biological parts

First, we computationally divided the whole yeast genome into genes and intergenic regions. We define three biological parts based on the structure of a eukaryotic gene, which is usually composed of a promoter (PRO), an open-reading frame (ORF) and a terminator (TER). We defined an ORF based on the coding regions from ATG start codon to the translational stop codon. A PRO is defined as 500 bp or up to the last gene boundary from the ATG codon of its ORF, whichever is shorter. Similarly, a TER is defined as 200 bp downstream or up to the next gene boundary from the stop codon of its ORF, whichever is shorter. A computational program was developed to automatically carve out these parts according to the design principles and genome annotation. The program also automatically designs primers which contain the appropriate prefix and suffix for each part to standardize them.

Meanwhile, we designed three part vectors to host the PRO, ORF and TER, respectively. These vectors are derived from a plasmid containing a kanamycin-resistance gene with the multiple cloning sites (MCS) replaced by a RFP reporter gene under the control of the bacterial *lac* promoter and *rrnB* T1 terminator. The *E. coli* colonies carrying these plasmids will display red pigment, which can be visually distinguished to facilitate clone identification. Three pairs of overhangs were chosen as the prefix and suffix respectively, including ‘ACCT-GATG’ for PRO, ‘GATG-TAGC’ for ORF and ‘TAGC-TGCC’ for TER. These were tested in advance and shown to yield high cloning efficiency (Table 2). In order to permit the precise insertion and release of each part with fixed overhangs, we designed a pair of BsaI and BsmBI restriction sites to flank the RFP reporter in such a way that both enzymes will generate the exact same overhangs, i.e. the designed prefix and suffix (Figure 1C and Table 1). Therefore, BsaI can be used for the construction of the part libraries and BsmBI can be used subsequently to release parts and assemble the TUs.

**Table 1.** The prefix and suffix sequences of the standard parts^a^

Vectors	Prefixes	Suffixes
HCKan_P	CGTCTCgGGCTaGAGACC	GGTCTCtGATGcGAGACG
HCKan_O	CGTCTCgGATGaGAGACC	GGTCTCtTAGCcGAGACG
HCKan_T	CGTCTCgTAGCaGAGACC	GGTCTCtCCTCcGAGACG
POT1	GGTCTCtACCTggctaGAGACG	CGTCTCacctcTGAGaGAGACC
POT2	GGTCTCtACCTggctaGAGACG	CGTCTCacctcAGGCaGAGACC
POT3	GGTCTCtAGGCggctaGAGACG	CGTCTCacctcTGAGaGAGACC
POT4	GGTCTCtAGGCggctaGAGACG	CGTCTCacctcTGGCaGAGACC
POT5	GGTCTCtTGCCggctaGAGACG	CGTCTCacctcTGAGaGAGACC
POT6	GGTCTCtTGCCggctaGAGACG	CGTCTCacctcCACTaGAGACC
POT7	GGTCTCtCACTggctaGAGACG	CGTCTCacctcTGAGaGAGACC
POT8	GGTCTCtCACTggctaGAGACG	CGTCTCacctcGTCGaGAGACC
POT9	GGTCTCtGTCGggctaGAGACG	CGTCTCacctcTGAGaGAGACC
POT10	GGTCTCtGTCGggctaGAGACG	CGTCTCacctcGGAGaGAGACC
POT11	GGTCTCtGGAGggctaGAGACG	CGTCTCacctcTGAGaGAGACC

^a^The recognition sites are underlined using the solid line for BsmBI and dashed line for BsaI. The 4-base overhangs released after digestion are shown in bold upper case.

**Table 2.** The efficiency of part assembly^a^

Parts	Plate ID	PCR amplification of parts from gDNA (%)	Confirm insertion by colony PCR (%)
PRO	ChrI_1_PRO	90.6	84.3
	ChrIII_1_PRO	91.7	91.7
	ChrIII_2_PRO	74.0	90.6
	ChrVII_2_PRO	97.9	82.3
	ChrVII_3_PRO	84.4	91.7
	ChrVII_4_PRO	84.4	78.1
	ChrVII_5_PRO	94.8	79.2
	ChrVIII_2_PRO	70.8	68.8
	ChrVIII_3_PRO	85.4	72.9
	ChrXII_1_PRO	88.5	85.4
	ChrXII_3_PRO	96.9	82.3
	ChrXII_4_PRO	94.8	87.5
	ChrXII_5_PRO	83.3	87.5
	ChrXII_6_PRO	99.0	93.8
ORF	ChrI_1_ORF	59.4	77.1
	ChrXII_1_ORF	77.1	70.8
TER	ChrI_1_TER	87.5	96.9
	ChrXII_1_TER	95.8	93.8
Average		86.4	84.2

^a^The efficiency was calculated based on a single round of assembly and listed as the percentage of positive PCR at the expected size. Only the plates with 96 pairs of primers were used in the calculation.

The efficiency of cloning for each part was tested in three steps. First, five known promoters were selected: pTEF1, pTEF2, pADH1, pTDH3 and pCYC1. We performed the one-pot assembly using cleaned PCR products. Four white clones were randomly isolated from the transformants and subjected to colony screening, plasmid preparation and restriction enzyme digestion. We found that almost all of the isolated clones were correct, with efficiency over 95%, suggesting that the Golden-Gate cloning method worked well. To reduce the labor in the process, we next tested whether it is possible to use PCR mixtures for assembly directly. We were able to obtain nearly 100% white bacterial colonies on the selective plates. However, unfortunately, none of these white clones contained the correct sequences, although dozens of colonies were tested (data not shown). DNA sequencing data revealed that only a very small DNA fragment was inserted into the vector, presumably from the primers in the PCR mixture. We also tried to optimize the PCR reaction by reducing the amount of polymerase and decreasing the amount of primers, but none of these reactions gave us high efficiency. Thereafter, we kept the PCR clean-up step in all of the future assembly protocols. At last, in order to test whether this protocol can be scaled up, we performed the assembly process in 96-well plates. Table 2 lists the efficiency of part amplification from genomic DNA and colony PCR to confirm the correct cloning using this protocol. Over 80% of the parts can be cloned in one pass on average. At the time of writing, over 2000 PROs have been cloned and sequence-verified.

One limitation to use of the Golden-Gate cloning method is that the same restriction sites may occur within a given sequence. To overcome this problem, two strategies could be adopted. One is to re-design parts to eliminate the internal sites, for example by codon optimization of an ORF, and the other approach is to leave the internal site intact as long as it does not generate the same overhangs used in the assembly method. In order to test how sensitive our protocol is to internal restriction enzyme sites, we chose four different parts, each containing one internal BsaI site within the sequence. We found that different proportions of red colonies appeared on the selective plates and on one plate no white colonies could be identified. From eight white clones we randomly tested from the remaining three reactions, we obtained 5 (62.5%), 4 (50%) and 3 (37.5%) correct ones, respectively (Supplementary Figure S1). Furthermore, by introducing digestion-ligation cycles and performing additional ligation after heat-inactivating the restriction enzymes (see Materials and Methods section for details), we were able to obtain the correct clones for the last part (four out of eight colonies tested were correct) and meanwhile improved the assembly efficiency to 75%, 100%, and 87.5% for the other three parts, respectively (Supplementary Figure S1). Therefore, we concluded that the presence of an internal restriction enzyme site may reduce the cloning efficiency but its removal is not necessary to obtain the correct clones in most cases.

### Characterization of the PRO activity

**Figure 2.**
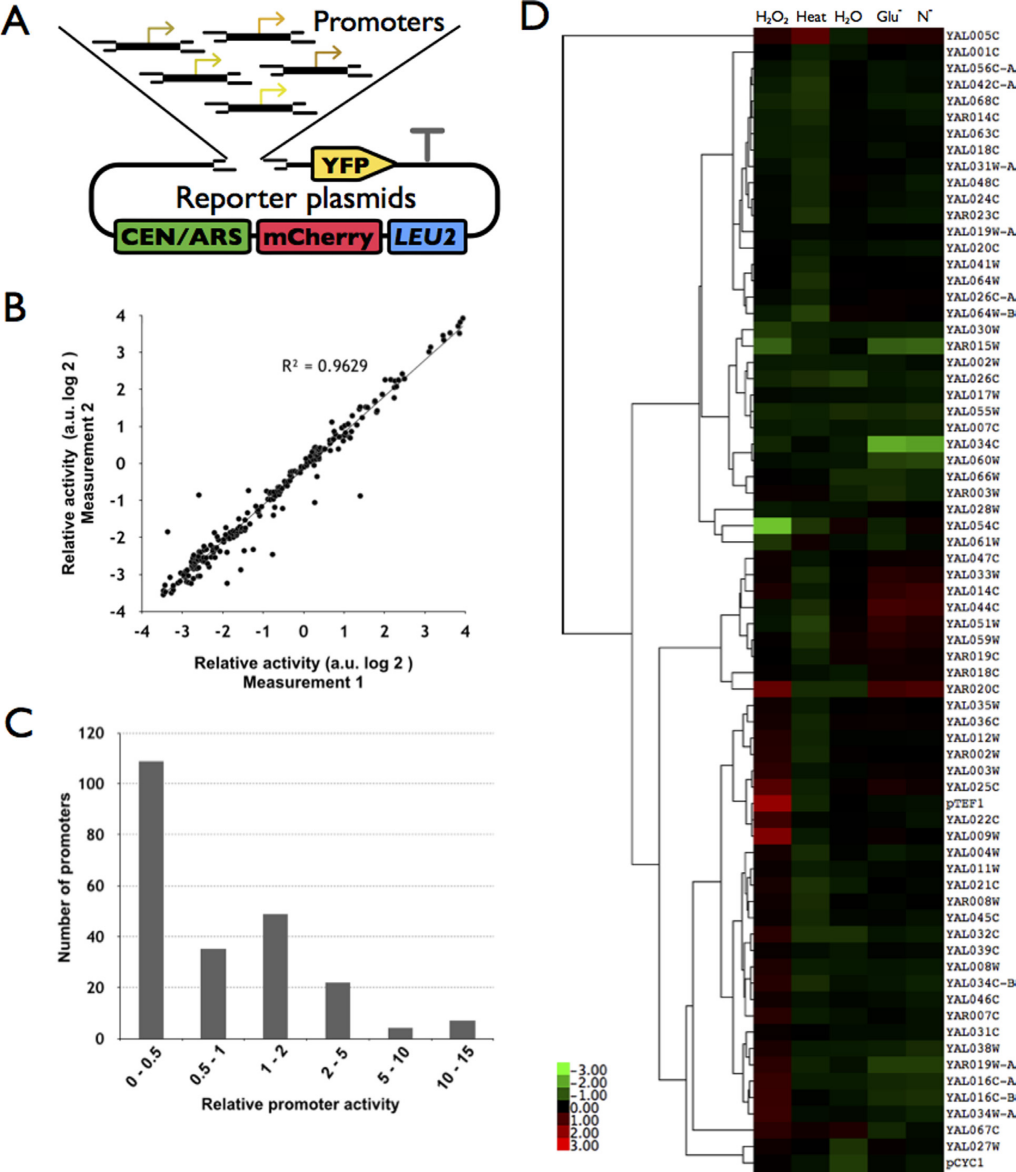
Characterization of the native promoters using a YFP-mCherry dual reporter system. (**A**) The YFP-mCherry reporter system. Each promoter was inserted upstream of the YFP gene in a plasmid, which also contains an mCherry fluorescent protein driven by the TEF2 promoter and terminator. The activity of a given promoter was defined as the ratio between the YFP and mCherry fluorescence intensity. (**B**) High correlation between repeated measurements (*R*^2^ = 0.96). Each dot represents a promoter, which was measured in two independent experiments. (**C**) The distribution of promoter activities. The strength of each promoter was normalized to that of CYC1 and categorized into six arbitrary groups. (**D**) Change of promoter activity under different stress conditions. The activity of each promoter was compared to that in the normal condition (in SC-Leu medium). Shown here is a total of 71 promoters, which were tested in the first batch of experiments.

In order to test the function of the constructed parts, we employed the same assay developed by Sharon *et al*. to measure the activity of PROs (32). Each PRO was inserted upstream of the YFP reporter gene to replace the RFP gene. Therefore, YFP fluorescence could represent the activity of the promoter at its native locus (Figure 2A). At the same time, the reporter plasmid contains an mCherry fluorescent protein gene under the control of the TEF2 promoter and terminator to serve as the internal control. As a proof of principle, reporter constructs for a total of 226 promoters from chromosome I and chromosome XII were constructed and characterized, and activity of each promoter is presented as the ratio of YFP and mCherry fluorescence intensities in each cell as detected by BD Fortessa cell analyzer (Supplementary Figure S2). We at first tested whether this measurement is reliable by multiple measurements at different time points and using independent colonies. Figure 2B illustrates high correlations between two repeated measurements at different time points or using two independent colonies, suggesting the measured activity is reliable. We found that the activity of these promoters ranges from very weak to very strong, but were not uniformly distributed, and most of them showed low activity (Figure 2C). It is possible that this particular set of promoters is not strong enough. Furthermore, in order to test how promoters behave under different conditions, we treated the cells with different stresses such as oxidative stress (H_2_O_2_), heat (37°C) and nutrient-starvation (in medium lacking glucose, nitrogen source or even just in water). We found that most of the promoters showed little change in activity, but behaved similarly under the different conditions, consistent with another study reported recently (36) (Figure 2D). The most obvious change was from the heat treatment, which leads to repression of many of the promoters. We expect to find some promoters that will be specifically activated or repressed under certain conditions once the sample size is large enough.

### Assembly of transcription units

**Figure 3.**
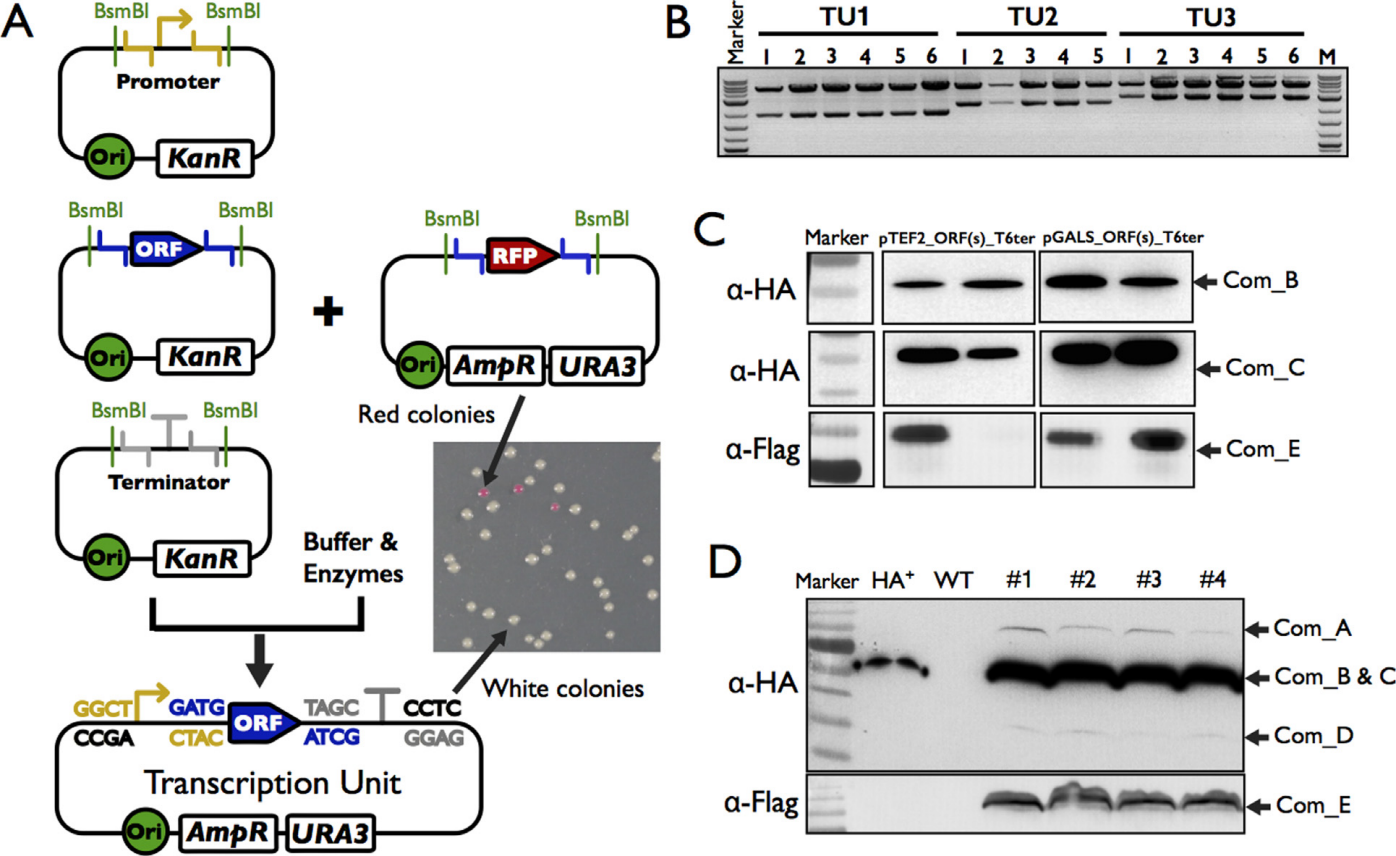
Assembly and functional testing of transcription units. (**A**) Schematic representation of the ‘One-POT’ assembly of transcription units. The standard parts (promoters, ORFs, terminators) and the ‘POT’ accept vector were mixed together with buffers and enzymes to assembly the transcription units in one tube. The red colonies are the residual intact acceptor vectors and the white colonies contain the correctly assembled transcription units. (**B**) Restriction enzyme digestion to confirm the assembled transcription units. Five or six white colonies were randomly picked for three different transcription units, and all of them showed the correct insert and vector at the expected size. TU1 is pTDH3-crtE-tTEF1 into POT2; TU2 is pADH1-crtI-tADH1 into POT4, and TU3 is pTEF2-crtYB-tTEF2 into POT5. (**C**) Western blotting to detect the expression of assembled TUs. Three human ORFs were tagged by HA or Flag epitope and assembled into the POT vectors under the control of TEF2 or GALs promoters. Two clones were randomly isolated after the plasmids were transformed into yeast. The expression of these proteins was detected using antibodies against HA or Flag tag. (**D**) Western blotting to detect the expression of a human complex integrated into the yeast genome. All five components are detectable using antibodies against the epitopes although the level of expression is not uniform.

In order to rapidly assemble transcription units from the biological parts, we designed two sets of vectors (designated as ‘POT’ vectors for PRO-ORF-TER) based on commonly used yeast high and low copy shuttle plasmids. These vectors were derived from the pRS plasmids (31) by eliminating the BsaI and BsmBI sites and replacing the multiple cloning sites (MCS) with a RFP gene similar to the one in the part vector, except that the position of the BsaI and BsmBI recognition sites was switched (Table 1). Therefore, it not only allows initial assembly of the parts, but also leaves them ready for the next round of assembly. The three donor plasmids and one POT vector were mixed together with enzymes and buffers, and the assembly was done using a similar protocol as that for part assembly (Figure 3A, see Materials and Methods section for details). The reaction mixture was directly transformed into bacteria to obtain correctly assembled targets.

To test the efficiency of TU assembly, we chose three sets of genes including three genes from the β-carotene synthesis pathway (37), five genes from the violacein pathway (38) and five genes from a human protein complex. The first two sets of genes were codon-optimized for yeast expression, synthesized *de novo* and cloned into the standard part vector. The last set of genes were either cloned into the standard part vector first or amplified from the target plasmids and used directly for the assembly. The yeast ADH1 terminator and various promoters such as pCYC1 and pTEF2 were used to create the TUs. Four white clones were randomly chosen from each reaction to confirm correct assembly. As shown in Table 3, we obtained 87.5%, 90% and 93.3% correct clones, respectively, indicating that our assembly protocol is highly efficient. The assembled TUs could be verified by digestion using restriction enzymes (Figure 3B). Furthermore, to demonstrate if the assembled genes were functional as expected, we used the epitopes on each of the five components of the human complex to confirm the expression of proteins. Figure 3C shows that when these clones were transformed into yeast cells, the proteins could be detected using antibodies against the epitopes. Finally, using the method described below, we assembled the five genes into the yeast genome, and western blotting indicated that all five proteins could be detected at varying expression levels (Figure 3D).

**Table 3.** The assembly efficiency of transcription units^a^

Assay method	β-Carotene synthesis genes	Violacein synthesis genes	Protein complex genes
Colony PCR	21/24 (87.5%)	18/20(90%)	28/30(93.3%)
Digestion	100%	100%	100%

^a^The efficiency listed here is for parts without internal BsmBI sites. For those containing one or more internal sites, the efficiency decreased dramatically if the same protocol was used.

### Hierarchal assembly of metabolic pathways

**Figure 4.**
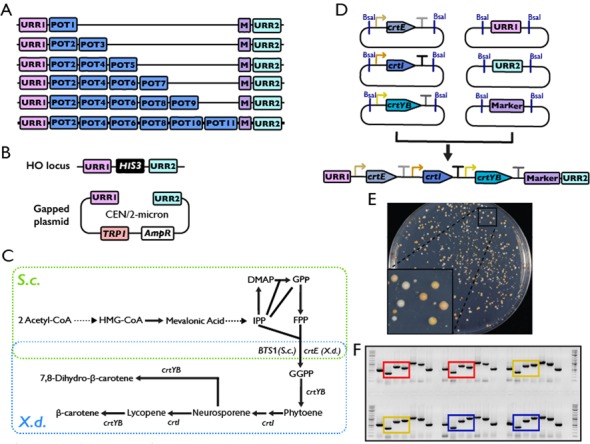
Hierarchical assembly of metabolic pathways. (**A**) Graphical representation of assembling a pathway with up to six genes by cherry-picking the eleven POT vectors. Different POT vectors should be used based on the number of genes to be assembled. (**B**) Different targets designed to accommodate the exogenous pathways. One is at the HO locus on chromosome IV, where a selectable marker (HIS3 as shown) flanked by the URRs is engineered. The other is an ectopic plasmid, either at low copy (CEN plasmid/pRS414-URR) or at high copy (the 2-micron plasmid/pRS424-URR). (**C**) Overall scheme for engineering β-carotene production in *S. cerevisiae*. Genes from *X. dendrorhous* are circled in blue. IPP, isopentenyl diphosphate; DMAP, dimethylallyl diphosphate; GPP, geranyl diphosphate; FPP, farnesyl diphosphate; GGPP, geranylgeranyl diphosphate. (**D**) Hierarchical assembly of β-carotene biosynthesis pathway. The assembled TUs (from Figure 3) in the POT vectors and URR1/URR2/Marker were digested with restriction enzymes, and ligated *in vitro* to the DNA fragment containing the complete pathway, which was used to transform the yeast directly. (**E**) The *S. cerevisiae* strain growing on the selective plate after transformation. The presence of yellow colonies indicates the cells could produce β-carotene successfully. The plate was photographed after 60 h of incubation at 30°C. (**F**) The diagnostic PCR of the *S. cerevisiae* transformants. Those circled in red are the cells containing URR1-pCYC1-crtE-tADH1-pTEF2-crtI-tADH1-pCYC1-crtYB-tADH1; those circled in yellow are the cells containing URR1-pCYC1-crtE-tADH1-pTEF2-crtItADH1-pTEF2-crtYB-tADH1; and those circled in blue are the cells containing URR1-pCYC1-crtE-tADH1-pTEF2-crtI-tADH1-pTDH3-crtYB-tADH1.

Usually, a metabolic pathway is composed of more than one gene. Most of the currently available methods are limited to testing only one gene at a time, and multiple plasmids have to be co-transformed into the host strain, which is time-consuming and impractical in terms of selecting for multiple plasmids in an industrial setting. Sometimes more than ten genes have to be assembled and tested, but the number of selective markers is limited, which makes this almost impossible. To overcome this limitation and achieve highly efficient assembly simultaneously, we designed eleven compatible POT vectors, which could allow us to perform hierarchical assembly of up to five component pathways in one step (Figure 4A and Table 1). A combination of these POT vectors can be chosen at the time of experiments depending on how many genes one needs to assemble, and the number of POT vectors could also be expanded to accommodate larger pathways. Alternatively, we can perform multiple rounds of assembly to keep expanding the pathway.

Two different targets were designed to accommodate the exogenous pathways (Figure 4B). One is at the HO locus on chromosome IV, where in the host strain we engineered a unique target site including a selective marker flanked by two unique recombination regions (URRs). Each URR is 500 bp in length, generated by a random sequence generator with 50% GC content. They have no homology to any known sequences in the NCBI database at the time of design (http://blast.ncbi.nlm.nih.gov/Blast.cgi). The incorporation of these URRs generated a specific target for the exogenous pathway. Once released from the POT vectors, the TUs could be ligated to the two URRs and a selective marker *in vitro*, forming a long DNA fragment with all TUs and the marker flanked by the two URRs (hereafter referred as the integration fragment, Figure 4D). Through homologous recombination using URRs, the assembled pathway could be integrated into the target chromosome, replacing the pre-existing selective marker at the locus. This allows us to quickly isolate correctly assembled yeast clones by simply selecting for autotrophic markers. The candidate clones could be further confirmed by diagnostic PCR to amplify regions spanning the junction between TUs.

The other target of the assembled pathways is in an ectopic plasmid, either at low copy number (CEN plasmid) or at high copy number (the 2-μm plasmid). This can be achieved by gap-repair, which operates at high efficiency in the budding yeast. Two types of receiving plasmids, derived from the yeast shuttle vectors pRS41X and pRS42X, were constructed by inserting the two URRs at the multiple cloning sites. The integration fragment described above and the linearized receiving plasmid can be co-transformed into a yeast strain to generate the assembled plasmids.

In order to test the system, we chose β-carotene biosynthesis pathway from *Xanthophyllomyces dendrorhous* as an example (Figure 4C). Budding yeast is able to make FPP. Conversion of FPP to β-carotene requires *crtE* (GGPP synthase), *crtI* (phytoene desaturase) and *crtYB* (bifunctional phytoene synthase and lycopene cyclase).(37,39) Each gene was cloned into a Part vector, assembled into a POT vector with different promoters but the same *ADH1* terminator, and subjected to Pathway assembly, directly targeting to the URR1-HIS3-URR2 at the HO locus. Figure 4E shows that after transformation, many orange colonies appeared, indicating the presence of carotenoids in these cells. After replicating the plate onto different selective plates, we isolated over a dozen colonies with expected markers, i.e. LEU+ HIS−, to confirm that the three genes were integrated correctly at the target locus by PCR (Figure 4F). We found that the correlation between positive PCR results and correct autotrophic markers is very high (Table 4), suggesting that this strategy works well and can save us the labor-intensive PCR verification process. However, we noticed that the number of clones with correct markers was not very high (10 in 360). In addition, there were many orange clones which could still grow on medium lacking histidine. It is possible that the pathway was integrated into the genome but somehow did not replace the target loci, or it existed in the cells in the form of plasmid(s). We reasoned that the low efficiency of obtaining phenotypically correct clones could partially come from the presence of the same terminator in each construct. Therefore three different terminators (*ADH1, TEF1* and *TEF2* terminator, respectively) were used to repeat the experiment. As expected, after transformation and replication, we found a great improvement in efficiency (Table 4).

**Table 4.** The efficiency of pathway assembly^a^

Promoters	Terminators	# of total clones (LEU+)	# of clones (LEU+URA-HIS-)	Confirmed by PCR
Individual	Same	13701	160 (1.17%)	54/56 (96.43%)
	Different	170	89 (52.35%)	6/6 (100%)
Pooled^b^	Same	1112	41(3.69%)	3/4 (75%)
		2416	76(3.15%)	13/13 (100%)
		32	8(25%)	6/7 (85.7%)
	Different	160	69 (43.13%)	20/20(100%)
		177	49 (27.68%)	
		199	60 (30.15%)	
		231	62 (26.83%)	
		229	65 (28.39%)	

^a^The efficiency listed here is for pathways assembled *in vitro* and integrated into the yeast genome directly.

^b^A total of 151 promoters were pooled together.

### Optimize β-carotene production using combinatory assembly method

With the designed part and POT vectors, we are able to quickly assemble an exogenous pathway such as the aforementioned β-carotene biosynthesis pathway in *S. cerevisiae*. Next, we investigate if these parts could be used to optimize the production of β-carotene.

**Figure 5.**
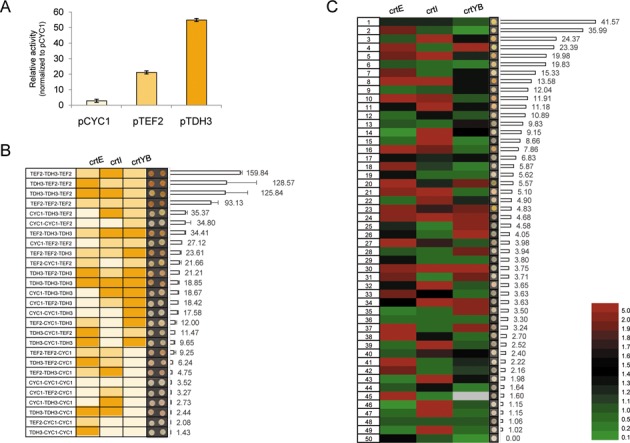
Optimize β-carotene production using a combinatorial assembly method. (**A**) The activity of three promoters CYC1, TEF2 and TDH3 was measured in our reporter system. The three promoters were chosen to represent the weak (blue), medium (green) and strong (red) promoter, respectively. (**B**) The production of β-carotene using different combination of the three promoters. The promoters were listed at the left most and color-coded as that in (A). Two colonies were spotted and the amount of β-carotene in each clone was quantified. (**C**) A total of 151 promoters were used to drive expression of each gene to optimize the production of β-carotene. Fifty clones, which contain the complete pathway, were randomly isolated. The identity of the promoter in front of each gene was determined and color-coded based on their activities for each isolates. The corresponding β-carotene content was quantified. The cells colored in grey indicate the identity of the promoter has not been determined. The colonies from (B) and (C) were grown on SC-Leu plates for 48 h with the starting optical density (OD_600_) being 1.

Three promoters, *pCYC1, pTEF2* and *pTDH3*, which represent the weak, medium and strong promoter respectively (Figure 5A), were used to drive expression of the three genes. To simplify the comparison and eliminate potential cross effect from terminators, the same *ADH1* terminator was used in all constructs. A total of twenty-seven possible combinations were assembled and tested in parallel (Figure 5B). Two clones containing the successfully assembled pathway from each combination were isolated and the amount of β-carotene in the cells was quantified. As shown in Figure 5B, the color of the colonies could be used as an rough indicator on the amount of β-carotene in the cell, which correlates well with the quantification result. Immediately, we found that the weakest promoter pCYC1 was completely excluded from the high β-carotene producing strains, suggesting that all three genes have to be highly expressed. Furthermore, once the *crtYB* gene, which catalysed the formation of phytoene from GGPP and β-carotene from lycopene, was under the control of a weak promoter, the production is greatly reduced. However, its expression should not be too high (when it is under the control of the strongest promoter, the production is also limited presumably due to the excessive metabolic burden to the host cells). Therefore, the expression of this gene should be carefully controlled in order to achieve high level of β-carotene production.

Next, we explored if we can identify an optimal combination of promoters that will allow the cells to express each enzyme to the ideal level autonomously and produce maximum amount of β-carotene. Using the first batch of promoters we made, which includes 151 promoters from ChrI and ChrXII, we constructed three pools of plasmids in which the *crtE, crtI* and *crtYB* were under the control of different promoters. This could allow us to generate over three million combinations of the three-gene pathway theoretically. We randomly isolated 50 clones that contain the full pathway and quantified the amount of β-carotene. At the same time we identified the promoters for each of the three genes in these clones by DNA sequencing. Shown in Figure 5C are the 50 clones sorted according to the amount of β-carotene. Since the strength of each promoter has been measured using aforementioned reporter assay, we color-coded the promoter activity. We found the β-carotene production in these clones varied in a broad range, with the peak areas ranging from none to over 2000 arbitrary units. However, the overall productivity is lower than what we could achieve using the three defined promoters. One possibility is that because most of the promoters used in this assay are weak promoters (Figure 2C), therefore, most assembled pathways did not yield high production of carotene compound. In the future, a subset of stronger promoters could be cherry-picked to avoid the use of a lot of weak promoters.

## DISCUSSION

Recent developments in synthetic biology and metabolic engineering gives hope for large-scale, inexpensive production of fuel, chemicals and materials using microorganisms (40). Extensive genetic engineering is required to modify the hosts’ genetic materials to allow the heterologous production of the desired chemicals, which generally demands proper expression of one or more critical metabolic enzymes. Unfortunately, precise and versatile tools to control the expression of these genes and metabolic pathways under both laboratory and industrial conditions are not fully developed, which in turn, lead to a long design, build, test and optimization cycle for every heterologous pathway (41).

To tackle this problem, we started by systemically designing standard natural biological parts in *S. cerevisiae*, which are currently not available for this eukaryotic model organism. At present, the available database on standard biological parts is from the Registry of Standard Biological Parts, which contains over 3000 parts, contributed from academic labs and student teams participating in the annual International Genetically Engineered Machine (iGEM) competition. Each part was designed with EcoRI and XbaI in the prefix and SpeI and PstI in the suffix, which allows two parts to be assembled together and regenerate the prefix and suffix (42,43). To make our parts compatible with theirs, we also incorporated a similar BioBrick Prefix and suffix in addition to the Type IIs restriction sites described in this paper. In addition, during the time when this manuscript was under reviewing, a report describing the yeast Golden Gate (yGG) assembly method was published in which a similar design of each part was adopted but with slightly different overhangs (44).

One requirement, which allows different research labs and companies willing to use these parts, depends on how convenient and robust these parts behave. As shown in Figure 3, the parts constructed in this study could be used easily through a one-pot reaction, before transforming into bacterium and the success rate is very high (near 100% when selecting the white clones). In addition, the classic ‘blue-white’ selection using LacZ as reporter needs special culture medium containing X-Gal to show the color (45,46), which will need extra cost and effort to make. In contrast, using our receiving vectors, the bacterium will display red color as long as an intact RFP genes are present in the cell, avoiding the use of any special type of medium. Furthermore, each part in our libraries will be sequence-verified to confirm its fidelity. On the top of that, the activity of every PRO will be measured, either individually or as a pool, using the reporter assay, which will be extremely useful to guide the users through the design process.

Generally, testing a heterologous pathway requires several steps including obtaining the candidate genes by PCR or gene synthesis, clone the genes into expression vectors (usually with arbitrary selected promoters and terminators) with different selective markers, transform the constructed genes into the host and test if an final production could be produced (47–49). During this process, one time-consuming step is to clone the genes, either individually into an expression vector or together to obtain a multiple-gene plasmid, which may take weeks to months. In addition, larger plasmid is usually hard to construct and could be potentially toxic to bacteria. To overcome this limitation, we designed each receiving vector compatible for subsequent round of assembly. For example, the part vectors are used to host each part for sequence verification and distribution. Each PRO, ORF and TER can be released and ligated together to form a TU by simply mixing the three plasmids and a POT vector in one tube followed incubation. Meanwhile, each assembled TU can be further assembled to form a multiple gene pathway *in vitro* and used to transform the host yeast strain directly. Through another commonly used plate-replicating technique, the strains containing the correctly integrated pathway could be easily identified. Therefore, our approach could simplify the testing process by avoiding the construction of larger plasmid (Figure 4).

Furthermore, as shown in Figure 4A, up to six TUs could be tested with eleven POT vectors. However, sometimes a pathway could contain a lot more TUs. There are two different approaches to meet this requirement using current system. One is to expand the number of POT vectors, which is disfavoured since it could be challenging to find large number of compatible 4-bp overhangs. In addition, with increased number of TUs in the *in vitro* ligation system, the efficiency to obtain the clones containing a complete pathway will drop significantly. The other approach is to carry out multiple rounds of the assembly processes. As shown in Figure 4D, after the first round of assembly, the UUR1-HIS3-URR2 cassette, for example, will be replaced by URR1-TU1-TU2-TU3-TU4-TU5-TU6-Marker1-URR2, which could serve as the target locus for another round of assembly. Next, a new set of TUs including the last TU, i.e. TU6, will be assembled together to generate the fragment TU6-TU7-TU8-TU9-TU10-TU11-Marker 2-URR2 *in vitro*. Through homologous recombination between TU6 and URR2, up to 11 TUs could be assembled at the target locus in two rounds. Theoretically, this process could be repeated unlimited times by swapping the two markers and generate a cluster of TUs as many as required.

While it is time-consuming to assemble and test a pathway in a heterologous host, it is more challenging to optimize a multiple-gene pathway. Usually, people used selected promoters, RBSs and terminators to overexpress every gene in the pathway. However, this strategy did not always lead to the maximum yield. Recently, quite a few studies are trying to optimize the production of a product thought manipulating expression of each gene in the pathway, so they can cooperate and produce the highest titre. For example, John Deuber *et al*. used five promoters to optimize the expression of five genes involved in violacein biosynthesis and recently eight genes involved in xylose utilization simultaneously and were able to identify strains with dramatically improved performance (38,50). More recently, the Voigt group demonstrated that combinatory method could also be applied to much larger biosynthesis pathways such as the 16-gene nitrogen fixation pathway to modulate the expression of multiple genes simultaneously (51). As presented in Figures 4 and 5, the part libraries allow a metabolic pathway could not only be quickly assembled but also be optimized by making large scale pools of TUs in which each gene is under the control of different PROs. The same strategy could be applied to other pathways to quickly optimize the production of certain natural products in *S. cerevisiae*.

In summary, we presented here the standard part libraries and assembly methods which could allow us to perform the construction of a complete pathway in as short as a week, and therefore provide researchers in metabolic engineering field a strategy to test and optimize a new heterologous pathway in *S. cerevisiae* quickly and cost-effectively.

## AVAILABILITY

The code to carve out YeastFab parts is available at Github (https://github.com/IsaacLuo/GenomeCarverPerl). All plasmids are in the process of depositing to addgene (71893) and will be available soon.

## SUPPLEMENTARY DATA

Supplementary Data are available at NAR Online.

SUPPLEMENTARY DATA
