# Supplementary material for: YeastFab: the design and construction of standard biological parts for metabolic engineering in Saccharomyces cerevisiae
Source: Nucleic Acids Res. 2015 May 8;43(13):e88. doi: 10.1093/nar/gkv464 (PMC4513847; doi:10.1093/nar/gkv464)
Supplement: SUPPLEMENTARY DATA [file supp_43_13_e88__index.html]

YeastFab: the design and construction of standard biological parts for metabolic engineering in Saccharomyces cerevisiae — YeastFab: the design and construction of standard biological parts for metabolic engineering in Saccharomyces cerevisiae — SUPPLEMENTARY DATA 

# YeastFab: the design and construction of standard biological parts for metabolic engineering in *Saccharomyces cerevisiae*

## SUPPLEMENTARY DATA

- SUPPLEMENTARY DATA
- SUPPLEMENTARY DATA
- SUPPLEMENTARY DATA
- SUPPLEMENTARY DATA
