# Supplementary material for: YeastFab: the design and construction of standard biological parts for metabolic engineering in Saccharomyces cerevisiae
Source: Nucleic Acids Res. 2015 May 8;43(13):e88. doi: 10.1093/nar/gkv464 (PMC4513847; doi:10.1093/nar/gkv464)
Supplement: SUPPLEMENTARY DATA [file supp_gkv464_nar-00787-met-h-2015-File007.docx]

**SUPPLEMENTARY DATA**


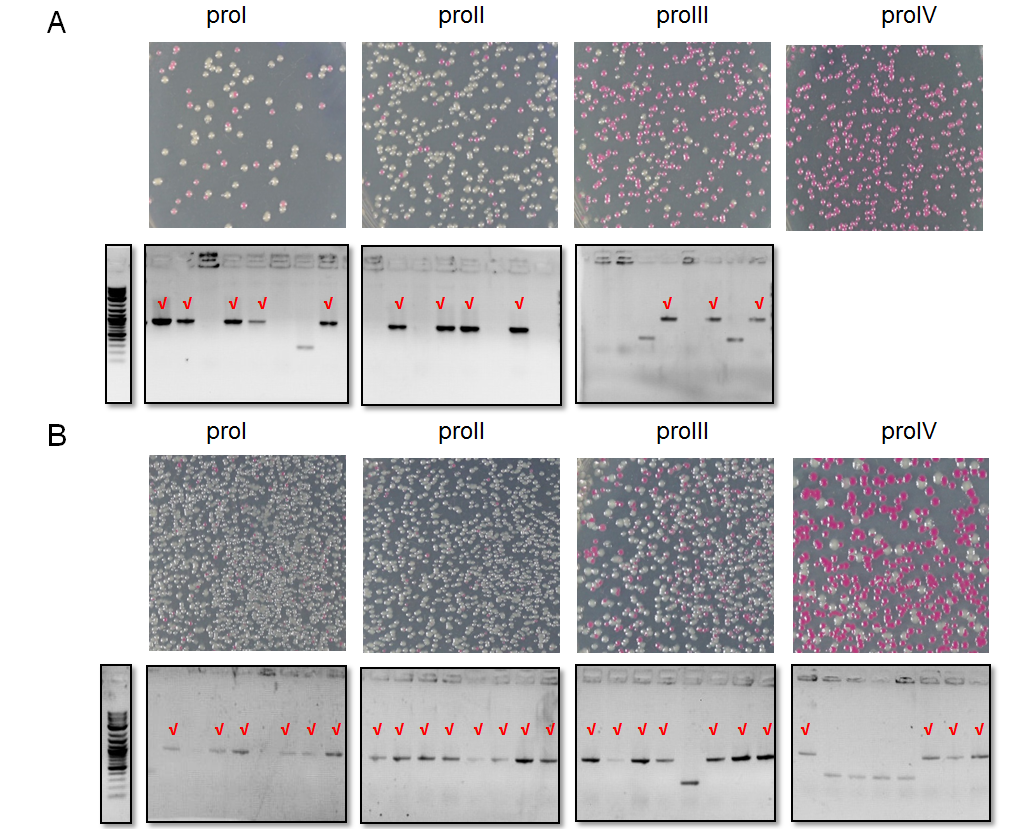


**Supplementary Figure S1.** The effects of internal RE site on the cloning efficiency of biological parts. Promoter I, II, III and IV, each contains 1 BsaI site in the middle, were used for assembly. Reactions in A were performed following standard protocol. Promoter IV didn’t generate any white clones, and therefore was excluded from PCR analysis. Reactions in B used the optimized protocol (See Material and Methods for details). In both A and B, the upper panels showed the bacteria colonies on selective plates after transformation, and the lower ones were results from colony PCR of eight randomly-picked, white colonies for each reaction.


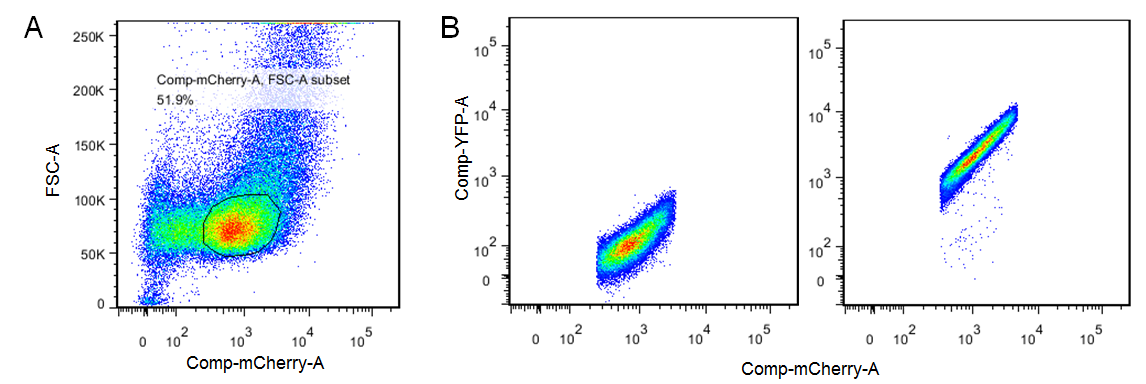


**Supplementary Figure S2.** Flow cytometry analysis of the cells containing the reporter plasmid. A. Cells with positive mCherry fluorescence signal and correct cell size (FSC signal) were gated for further analysis. B. Representative images of cells with lower or higher expressed YFP (which represents weak or strong promoters)
